# Supplementary material for: Iron Accumulation and Changes in Cellular Organelles in WDR45 Mutant Fibroblasts
Source: Int J Mol Sci. 2021 Oct 28;22(21):11650. doi: 10.3390/ijms222111650 (PMC8584078; doi:10.3390/ijms222111650)
Supplement: Supplementary file 1 [file ijms-22-11650-s001.zip › ijms-1341312-supplementary.pdf]

Supplementary Information

Iron accumulation and changes of cellular organelles in WDR45 mutant fibroblasts

Hye Eun Lee[1,†], Minkyo Jung[1,†], Seulgi Noh[1], Hyebin Choi[2], Sehyun Chae[2*], Jae-Hyeok Lee[3,4*], Ji Young Mun[1*]

[1]Neural circuit research group, Korea Brain Research Institute, Daegu, Korea, [2]Neurovascular unit research group, Korea Brain Research Institute, Daegu, Korea, [3]Department of Neurology, Research Institute for Convergence of Biomedical Science and Technology, Pusan National University Yangsan Hospital, Yangsan, Korea, [4]Medical Research Institute, Pusan National University School of Medicine, Yangsan, Korea

Materials and Methods

Cell viability assay.

Cell viability was determined using Cell Counting Kit-8 (CCK-8, Dojindo Laboratories, Japan) according to the manufacturer's instructions. Briefly, cells were seeded in 96-well plates at a density of 2×10^3 cells per well. The cells were allowed to adhere for 24, 48 and 72 hours. The OD 450 values were measured, and the growth curve was plotted. The experiments were repeated three times.

ROS assay.

The intracellular content of reactive oxygen species (ROS) was determined by measuring the fluorescence intensity of 2′, 7′-dichlorofluorescein (DCF). Cells were grown in 35 mm glass bottomed culture dishes (NEST Biotechnology Co., Wuxi, China, 801001) to 60 - 70 % confluency. Next day, to visualize ROS, H_2DCFDA (2',7'-dichlorodihydrofluorescein diacetate, Thermo Fisher Scientific, Waltham, MA, USA, D399) was added to the cell medium at a final concentration of 10 μM and incubated at 37°C for 30 minutes. The cells were then washed carefully with phosphate-buffered solution. Immediately, cells were observed under a confocal light microscope (Ti-RCP, Nikon, Japan). For Iron scavenger, cells were treated with iron chelator Bpy (2,2'-bipyridine) a final concentration of 1 mM and the cells were further incubated at 37°C for 15 hours. Fluorescence intensity was measured using ImageJ software.

mRNA quantification

For quantitative real-time reverse-transcription PCR (RT-qPCR), RNA was harvested using TRIzol lysis buffer (Invitrogen, Carlsbad, CA, USA), followed by DNase I treatment, and first-stand cDNA synthesis was performed using 1ug of total RNA and oligo(dT) for reverse priming with SuperScript III Supermix (Invitrogen, Carlsbad, CA, USA). Amplification Real-time PCR was performed using a SYBR Green PCR Master Mix (Applied Biosystems, Foster City, CA, USA). Each PCR reaction contained cDNA at a 10-fold dilution, and gene-specific primers. The thermal cycle used was 2 min at 50℃, 10 min at 95℃, 40 cycles of 15 sec denaturation at 95℃ with 1 min annealing at 60℃. The mean cycle threshold (CT) values were calculated, with normalization to GAPDH as an internal control. Samples were analyzed using quantitative real-time PCR with gene-specific primer pairs, on a ABI 7500 fast real-time PCR detection system (Life Technologies, Foster City, CA, USA) using the ΔΔCT method [29]. In each case, multiple reactions were performed using two to three independent biological replicates, with the primers listed in Supplementary Table S3.

Supplementary Figures

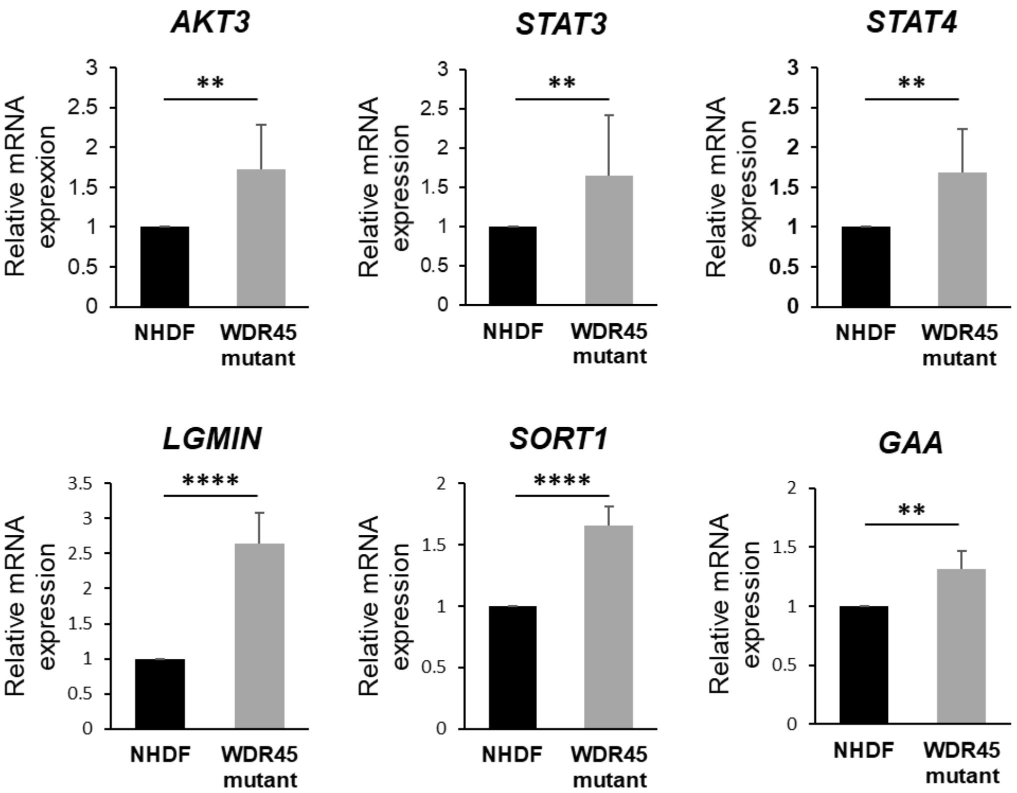

Supplementary Figure 1. Relative mRNA levels of the upregulated genes in WDR45 mutant fibroblasts were analyzed by quantitative RT-PCR. mRNA level of each gene was normalized using the GAPDH level. Data are shown as the mean ± SD (n = 5-8). **p < 0.01, ****p < 0.0001 by Student's t-test.

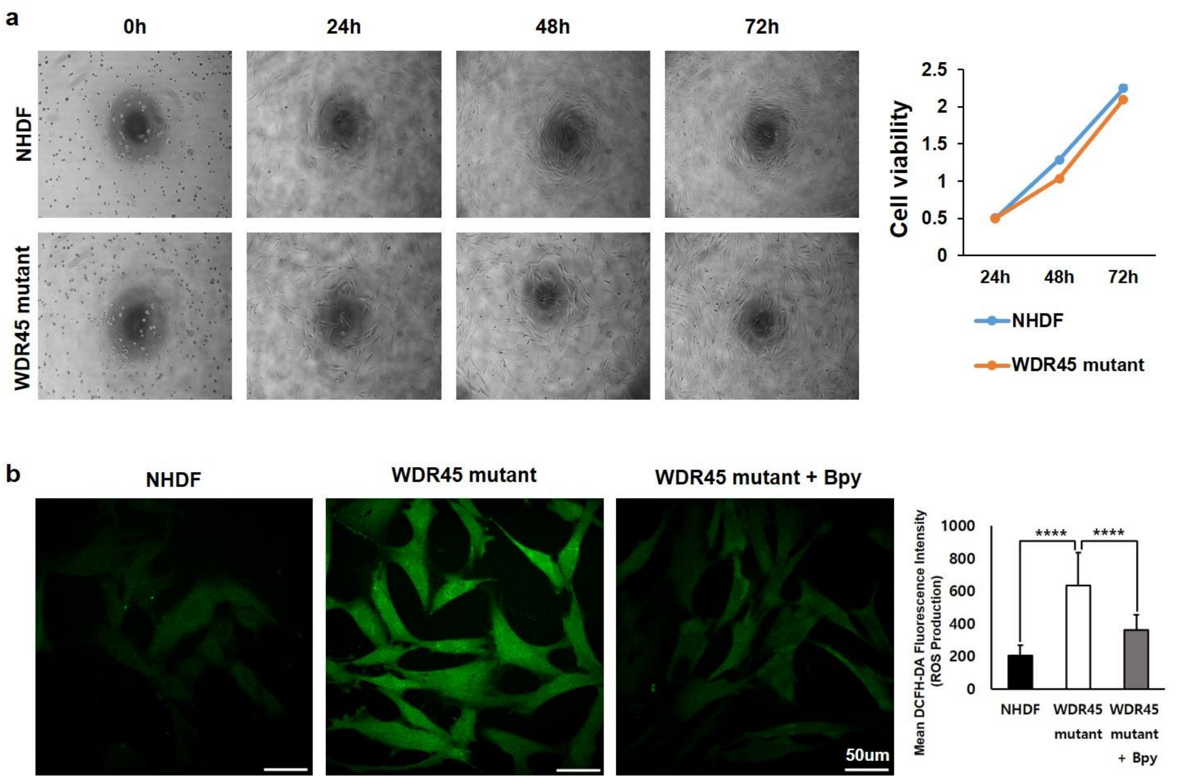

Supplementary Figure 2. Cell proliferation was measured by CCK-8 cell viability assay (a), and Detection of ROS by fluorescence intensity measurement using a fluorescence microscope. Intracellular ROS levels assayed with DCFH-DA fluorescent probe treated with or not iron scavenger (Bpy : 2,2′-Bipyridyl) (b). Data are shown as the mean ± SD (n = 90). ****$p < 0.0001$ by Student's t-test.

Supplementary Tables

Supplementary Table 1. 2,639 differentially expressed genes (DEGs). Ensembl IDs, Entrez IDs, symbols, descriptions, P-values, and log2-fold-changes of the DEGs are shown. P-values were computed as described in Materials and Methods section. (See the attached excel file**)**

Supplementary Table 2. mRNA seq results. The numbers of the total reads, unique mapped reads, and mapped reads were shown. UMR represents the number of reads uniquely mapped. '% mapped reads' means the percentage of the mapped reads (UMR and multi-mapped reads) over the total sequenced reads.

Sample	Total reads	Unique mapped reads (UMR)	Mapped reads (unique + multiple mapped reads)	% mapped reads
NHDF-1	69,983,503	65,091,735	66,931,921	95.6
NHDF-2	74,818,864	68,512,791	70,978,531	94.9
NHDF-3	78,123,920	73,040,694	75,135,689	96.2
WDR45-1	70,965,313	64,139,081	67,183,956	94.7
WDR45-2	76,182,187	70,074,959	72,824,837	95.6
WDR45-3	69,789,911	65,017,513	66,907,950	95.9

Supplementary Table 3. Primer information

Gene name		5′-3′
TFEB	Forward	CCTGGAGATGACCAACAAGCAG
	Reverse	TAGGCAGCTCCTGCTTCACCAC
ACP5	Forward	CATGACCACCTTGGCAATGTCTC
	Reverse	CTGTGGGATCTTGAAGTGCAGG
CTSD	Forward	GCAAACTGCTGGACATCGCTTG
	Reverse	GCCATAGTGGATGTCAAACGAGG
VAMP4	Forward	GCTTATCGGATAATGCAACAGCTT
	Reverse	GCAGCAACCAAAGCCATGATGG
PPARGC1A	Forward	CCAAAGGATGCGCTCTCGTTCA
	Reverse	CGGTGTCTGTAGTGGCTTGACT
SLC27A1	Forward	TGACAGTCGTCCTCCGCAAGAA
	Reverse	CTTCAGCAGGTAGCGGCAGATC
ACSL4	Forward	GCTATCTCCTCAGACACACCGA
	Reverse	AGGTGCTCCAACTCTGCCAGTA
CPT1C	Forward	GGATGGCACTGAAGAGGAAA
	Reverse	TCCTGGAAAAGGCATCTCTC
ACAT1	Forward	CACGCCTTTCACCAGTGGTGAC
	Reverse	GGCATTGGTGATCCAGGCTTTG
ACADS	Forward	CACGCCTTTCACCAGTGGTGAC
	Reverse	GGCATTGGTGATCCAGGCTTTG
AKT3	Forward	CGGAAAGATTGTGTACCGTGATC
	Reverse	CTTCATGGTGGCTGCATCTGTG
STAT3	Forward	CTTTGAGACCGAGGTGTATCACC
	Reverse	GGTCAGCATGTTGTACCACAGG
STAT4	Forward	CAGTGAAAGCCATCTCGGAGGA
	Reverse	TGTAGTCTCGCAGGATGTCAGC
LGMN	Forward	CCTGAAGATGGAGGCAAGCACT
	Reverse	GTTCGTCAGGAATCCCATTGCG
SORT1	Forward	ACTCTCAGAGCCGAATGCCGTA
	Reverse	CCTTCCAGCATCTTTGTCCAGG
GAA	Forward	TCTACAGCGTGGAGTTCTCCGA
	Reverse	GCTGAAGGAACTGGTCCGCAAA
